# Pulmonary Embolization After Gastric Varices Obliteration

**DOI:** 10.7759/cureus.49329

**Published:** 2023-11-24

**Authors:** Mafalda Duarte, Marta Lopes, Martim Trovão Bastos, Ana Monteiro, Rodrigo Duarte

**Affiliations:** 1 Internal Medicine, Hospital Prof. Doutor Fernando Fonseca, Amadora, PRT; 2 Internal Medicine, Hospital Egas Moniz, Lisbon, PRT

**Keywords:** acute gastrointestinal bleeding, alcoholic liver diseases, acute pulmonary embolism, n-butyl-2-cyanoacrylate, gastric varices

## Abstract

N-butyl-2-cyanoacrylate (NB2CYA) is frequently used in the treatment of variceal hemorrhage with a success rate in hemostatic control of 87%-100%. Although rare, complications include esophageal perforation, infection, or arterial and venous embolization. We present the case of a 67-year-old male with chronic ethanolic liver disease hospitalized due to melena and hematemesis. He had anemia requiring transfusion support, octreotide, and pantoprazole infusion. Upper digestive endoscopy was performed showing gastric varices with a hemorrhagic rupture point treated with cyanoacrylate. The patient developed respiratory failure over the next 48 hours with chest computed tomography (CT) angiography showing several dense, scattered linear images, with arterial vascular trajectories suggestive of cyanoacrylate embolization. It was decided to provide ventilatory support with invasive mechanical ventilation, initiate systemic corticosteroid therapy, and transfer the patient to the intensive care unit (ICU). The patient was ventilated for 11 days with initial favorable evolution, but after two episodes of decompensation of his chronic liver disease (CLD) (hepatic encephalopathy and hepatorenal syndrome) and a new nosocomial pneumonia, he ended up dying. The present case illustrates a rare but potentially fatal complication associated with cyanoacrylate, highlighting the importance of a high suspicion index in cases of respiratory failure and dyspnea after this therapy.

## Introduction

Gastrointestinal varices are dilated submucosal veins that are associated with portal hypertension and whose acute bleeding can have a fatal outcome. Bleeding from esophageal or gastric varices occurs very frequently in patients with decompensated chronic liver disease (CLD), increasing in frequency as the disease progresses. Around 20% of gastrointestinal variceal bleeding corresponds to gastric variceal bleeding [1], with a higher mortality rate than esophageal variceal bleeding, especially in patients with portal hypertension [1,2].

Currently, the approach to varices is based on prophylaxis of acute hemorrhage, preventing cirrhosis decompensation [1]. According to the Baveno VI Consensus, the first line of therapy for gastric varices is a combination of non-selective beta-blockers (NSBB) and N-butyl-2-cyanoacrylate (NB2CYA) injection. Some centers also use transjugular intrahepatic portosystemic shunt (TIPS) to control acute bleeding from gastric varices in patients at a high risk of failure [1,3].

Unfortunately, NB2CYA injection may have serious complications such as venous and arterial embolism (pulmonary embolism, cerebrovascular infarction, mesenteric infarction, and splenic infarction, among others), esophageal perforation, and sepsis [2-5].

The pathophysiology of pulmonary embolism associated with NB2CYA injection seems to be associated with a portosystemic high-flow vascular shunt, particularly a gastrosplenorenal shunt that reopens from embryonic vascular pathways, which also occurs in portal hypertension [3-5]. These portosystemic shunts may serve as a shortcut for NB2CYA embolization, which reinforces the need for angiographic studies prior to endoscopic intervention [3].

## Case presentation

We present the case of a 67-year-old male with a medical history of alcoholic chronic liver disease, with active alcohol consumption, and without any medical follow-up or medication for the last four years.

The patient was admitted to the emergency department (ED) with a week-long episode of nausea, coffee grounds vomiting, and dark, foul-smelling feces. Upon admission to the ED, he was hemodynamically stable, confused with a Glasgow Coma Scale (GCS) score of 14, and jaundiced, with non-tension ascites and evidence of black stools compatible with melena on anorectal examination.

The detailed laboratory evaluation is described in Table 1, with evidence of severe anemia, thrombocytopenia, and liver dysfunction with hyperbilirubinemia and increased international normalized ratio (INR) and hypoalbuminemia.

**Table 1:** Laboratory results pCO2: partial pressure of carbon dioxide, pO2: partial pressure of oxygen, HCO3: bicarbonate, mmol/L: millimoles per liter, mg/dL: milligrams per deciliter, g/dL: grams per deciliter, MCV: mean corpuscular volume, fl: femtoliters, WBC: white blood cell, μl/L: microliter per liter, INR: international normalized ratio, RCP: reactive chain protein, Cr: creatinine, ALT: alanine transaminase, AST: aspartate transaminase, LDH: lactate dehydrogenase, ALP: alkaline phosphatase, GGT: gamma-glutamyl transferase, μmol/L: micromoles per liter

Laboratory studies	Results	Reference values
pH	7.465	7.35-7.45
pCO2 (mmol/L)	23.7	35-45
pO2 (mmol/L)	70	80-100
HCO3 (mmol/L)	16.7	22-26
Lactate (mmol/L)	6.1	<1
Glycemia (mg/dL)	172	<120
Hemoglobin (g/dL)	5.6	12-14
MCV (fL)	123	80-100
WBC (μl/L)	13.4 × 10^9^	4.5-10 × 10^9^
Platelets (cells/L)	101,000	150,000-410,000
INR	1.7	1.2
Sodium (mmol/L)	131	135-145
Potassium (mmol/L)	5.85	3.5-5
RCP (mg/dL)	0.57	<0.5
Cr (mg/dL)	0.85	0.5-0.9
Urea (mg/dL)	98	<50
AST (U/L)	85	<32
ALT (U/L)	25	<33
LDH (U/L)	289	6-160
ALP (U/L)	862	36-150
GGT (U/L)	74	8-40
Albumin (g/dL)	2.86	3.5-5.4
Total bilirubin (mg/dL)	6.33	0.3-1.2
Ammonia (μmol/L)	50	<45

A nasogastric tube was placed without evidence of active or recent hemorrhage. The diagnosis of gastrointestinal bleeding and decompensated CLD was made, and the patient was hospitalized for stabilization and study.

Support was requested from the gastroenterology department, which suggested starting therapy with ceftriaxone 2 g, pantoprazole infusion 80 mg/hour, octreotide infusion 50 μg/hour, and red blood cell transfusion support to ensure a hemoglobin higher than 7 g/dL.

Regarding the etiological study, the patient underwent an upper digestive endoscopy, which revealed the presence of “large isolated gastric varices type 1 and esophageal varices of the Sarin classification with hemorrhagic points that were treated with an injection of NB2CYA.” An abdominal computed tomography (CT) angiography was also performed showing “moderate-large ascites, portal hypertension: dilation of the splenoportal axis, splenorenal shunt, dilation of the short and left gastric veins with large varices of the gastric fundus, the findings being related to phenomena of portal hypertension with portosystemic shunt pathways.”

**Figure 1:**
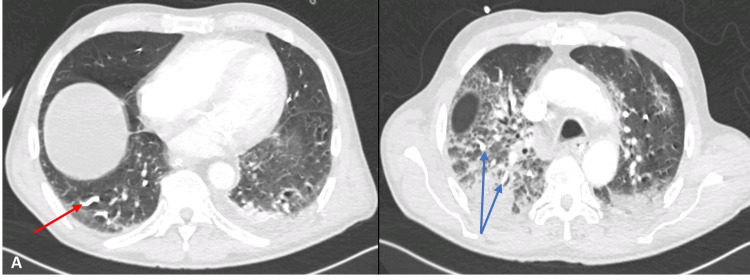
Chest CT scan showing embolization of NB2CYA A: 2 cm thrombus at subsegmental arteries (red arrow). B: Several thrombus and lung opacities (blue arrows). CT: computed tomography, NB2CYA: N-butyl-2-cyanoacrylate

The patient progressed unfavorably over the following 48 hours with severe acute respiratory failure requiring oxygen therapy at 15 L/minute by Venturi mask. The laboratory evaluation did not show an increase in inflammatory parameters, the viral respiratory panel (test for influenza and SARS-CoV-2) was negative, and the chest X-ray showed a new infiltrate in the right upper lobe. To clarify the situation, a chest CT was performed (Figure 1), which revealed “several linear densities, dispersed throughout both lungs, with arterial vascular paths that are most likely related to arterial embolization of NB2CYA. These clots are in both lungs, namely, upper lobes, right interlobar artery, middle lobe, lingula and lower lobes. The largest are around 2 cm and are found in segmental and subsegmental arteries. Evidence of dilation of the pulmonary artery caliber related to probable pulmonary hypertension.”

The diagnosis of pulmonary arterial embolism by NB2CYA was made, and the case was discussed with the gastroenterology department, the intensive care unit, and the transplant unit to outline the best approach. Considering a Child-Pugh C (14 points), Model for End-Stage Liver Disease (MELD) score of 20 points, the presence of hepatic encephalopathy and pulmonary hypertension, maintenance of alcohol consumption, and loss of medical follow-up, it was concluded that there would be no indication for liver transplantation, nor for TIPS. After reviewing the literature and taking into account the impossibility of removing the clots as they were fragmented and dispersed throughout both lungs, it was decided to carry out supportive therapy with corticosteroid therapy (methylprednisolone 40 mg/day) and ventilatory support. In this context, the patient ended up being intubated and ventilated and transferred to the ICU where he remained for 12 days.

During his stay in the ICU, he completed 12 days of corticosteroid therapy, and due to a progressive increase in inflammatory parameters and continued respiratory failure, he also completed seven days of antibiotic therapy with piperacillin-tazobactam, assuming nosocomial pneumonia. The patient evolved favorably and was extubated after 11 days of invasive mechanical ventilation, with progressive withdrawal of oxygen therapy up to 2 L/minute. Also, it is worth mentioning that during hospitalization in the ICU, he had several episodes of delirium and two episodes of CLD decompensation, one of which was due to hepatorenal syndrome requiring albumin and terlipressin.

The patient ended up being transferred to the gastroenterology department for continued care and optimization of CLD and its complications. After a few days, he evolved poorly with increased needs for oxygen, elevated inflammatory biomarkers, and radiological evidence of nosocomial pneumonia with no response to antibiotic treatment or respiratory support. Despite all efforts, the patient ended up dying.

## Discussion

N-butyl-2-cyanoacrylate, or just cyanoacrylate, is a synthetic monomer that when in contact with ionic substances (such as water or blood) polymerizes into a solid mass that obliterates the vascular lumen and stimulates local thrombosis. Some studies report a hemostatic efficacy of around 87%-100% [3]. A controlled trial demonstrated that NB2CYA injection had fewer bleeding complications and lower mortality when compared to NSBB [4].

Complications associated with NB2CYA injection include venous and arterial embolism, including pulmonary thromboembolism or stroke, splenic and portal vein thrombosis, esophageal perforation, and sepsis [2,3,5,6]. A retrospective review identified a risk of embolization associated with the injection of NB2CYA between 0.5% and 4.3% [6]. This embolization risk was related to some risk factors such as large varicose veins, presence of perisplenic shunts (even if unknown), high monomer volume, and higher speed of injection [3,6-8].

Regarding the treatment approach, the literature is controversial. Some authors defend a conservative approach with respiratory support and corticosteroid therapy and may consider the use of anticoagulation and/or thrombolysis. If the NB2CYA emboli are localized at a specific area, a more interventive approach with emboli removal may be used [3,5-8].

## Conclusions

NB2CYA pulmonary embolization is a rare and potentially fatal complication of endoscopic treatments whose diagnosis requires a high index of suspicion.

The present case illustrates a rare but potentially serious complication associated with the use of cyanoacrylate, thus highlighting the importance of a high index of suspicion in cases of respiratory failure and dyspnea after this therapy.

In the few cases described in the literature, therapy was supportive, with invasive mechanical ventilation in most cases and corticosteroid therapy. Although it is not consensual, some authors suggest anticoagulation, which was not considered in this case given the hemorrhagic condition. Further research on this topic is needed in order to identify the best approach to the complications of NB2CYA injection.
